# Contentious Connectivity—the USA, Japan, and the Free and Open Indo-Pacific

**DOI:** 10.1007/s12140-023-09407-7

**Published:** 2023-05-01

**Authors:** Bart Gaens, Ville Sinkkonen

**Affiliations:** Finnish Institute of International Affairs, Helsinki, Finland

**Keywords:** Connectivity, Global order, Regionalism, Great-power competition, Infrastructure, Sustainable development

## Abstract

This article explores how the USA and Japan have aimed to advance connectivity and infrastructure investment in the Indo-Pacific, implicitly or explicitly in response to China’s Belt and Road Initiative (BRI). Both actors’ vision, strategies, and policies have been rolled out under the banner of the “Free and Open Indo-Pacific” (FOIP). The article first frames connectivity and the FOIP construct in the context of regional order and great-power relations in the Indo-Pacific. It then provides an in-depth assessment of the different initiatives by the USA and Japan, scrutinizing their progress on the ground, shortcomings, and relevant interlinkages. After an analysis of the logics that inform these connectivity initiatives, the article offers three key axioms and assesses implications for order more broadly. First, the West must fix the gap that often exists between rhetoric and capabilities in the sphere of infrastructure investments. Second, Western actors, including the USA and Japan, need to be clear about objectives. Namely, they must decide whether the aim of connectivity is to compete directly with China or to focus on complementarities and comparative advantages. Third, the USA and Japan need to prioritize connections and spheres of connectivity that are deemed strategically central, at the expense of others. More generally, given the connective logics that key actors currently harness, a fracturing of the region into one of the different orders comprising competing yet overlapping connections beckons.

## Introduction

The recent “turn” to connectivity has been driven forward, above all, by China’s Belt and Road Initiative (BRI, 2013), which uses infrastructure development and connections in all forms to create interdependencies between China and other parts of Asia as well as Europe and Africa. Other key players have responded to China’s forays, seeking to balance against what they perceive as Beijing’s attempts to enhance its influence through connectivity by proposing their own strategies. This has resulted in numerous policy papers, initiatives, and an equal number of new acronyms to denote the strategies. Examples include the United States’ Blue Dot Network (BDN); Japan and India’s Asia Africa Growth Corridor (AAGC); the G7’s Build Back Better World (B3W) and Partnership for Global Infrastructure and Investment (PGII); the Quadrilateral Security Dialogue (Quad) between the USA, Japan, Australia, and India; and the EU’s Global Gateway (EUGG).

This article provides one analysis of how Western connectivity endeavours in the Indo-Pacific have unfolded so far by mining the connectivity approaches of the USA and Japan—the key “Western” connectivity players in the region. Both actors’ vision, strategies, and policies have been advanced under the banner of the “Free and Open Indo-Pacific” (FOIP), initially a Japanese idea to which the USA subscribed in 2017.^1^ In particular, the paper is built around four interrelated questions: What kinds of connectivity initiatives have the USA and Japan embarked upon? How have they fared in practice? How are the American and Japanese approaches interlinked? What logics of connectivity, as laid out in the theoretical framework of this special issue by Gaens, Sinkkonen, and Vogt [2], are present in American and Japanese conduct? By reflecting on these questions, the present article offers three policy axioms for a more sustainable American and Japanese approach to connectivity, and also considers broader implications for regional order(ing) in the Indo-Pacific.

To set the stage, the article begins with a brief exposition on the state of great-power relations, particularly in the Indo-Pacific, positing that the present era is marked by “strategic” or “great-power” competition between the USA and its partners, on the one hand, and China, on the other. It then illustrates that for the USA and Japan, the FOIP construct has functioned as the overarching rubric for engaging in this contest, and further argues that connectivity should be understood as a key site and means of this contest. Following this, we introduce the theoretical scaffolding for the discussion—drawing on Gaens, Sinkkonen, and Vogt’s [2] work—introducing the different connectivity logics utilized for analyzing American and Japanese connectivity strategies. These logics have constitutive or corrosive effects on (regional) order(ing), depending on how close they fall to the cooperative or coercive ends of the connectivity spectrum. The article then turns to a discussion of the connectivity forays of the USA and Japan, as well as their successes, shortcomings, and relevant interlinkages. This is followed by a deeper exposition on the logics that inform these connectivity initiatives. The article concludes with a discussion of policy axioms and implications for order more broadly.

## Great-Power Competition and the Free and Open Indo-Pacific

The current age in international politics is defined by increasingly tense great-power competition between the USA and its great-power challengers China and Russia across manifold spheres, whether military, economic, institutional, technological, or cyber [3]. In the case of US-China relations, this contest has unfolded most prominently in the realm of economics and technology [4], but has also gathered military overtones, most recently manifest in tensions across the Taiwan Strait.

On the one hand, this contentious dynamic is indicative of a gradual power transition in the international system, from uni- to bi- or multipolarity [5–7]. On the other hand, the great-power contest is unfolding over the parameters of international order, the institutional and social foundations of international politics that “guide the relations among states” [8:47]. China has sought to alter the Western-led liberal international order from within, bidding for leadership positions in key institutions like the UN Food and Agriculture Organization (FAO) and International Telecommunications Union (ITU) [9], as well as from without, setting up alternatives including the Asian Infrastructure Investment Bank (AIIB) and the Shanghai Cooperation Organization (SCO) [10, 11:ch.4]. Beijing has also made a concerted effort to fortify its central position in regional trade as a founding member of the Regional Comprehensive Economic Partnership (RCEP). In addition, China is pushing a sovereignty- and non-interference-based understanding of the international order, which more and more frequently clashes with the West’s emphasis on human rights, democracy, and freedom [12].

In Washington D.C., the imperative of competing with China has become a rare area of bipartisan common ground [13–15]. In fact, the considerable similarity in the China policies of the Trump and Biden administrations especially in the realms of trade, regional military posturing, and technology proves that this view has considerable purchase in policymaking circles and, increasingly, the voting public [16].

China’s connectivity strategy has been a central sticking point in great-power competition. China’s efforts in this field arguably started already around the turn of the century when Beijing initiated its “Going Out” policy, boosting the country’s Foreign Direct Investments (FDI). The efforts intensified in the early 2010s, culminating in the launch of the BRI in 2013, originally conceptualized as the Silk Road Economic Belt, which seeks to join China with Europe via land, and the Maritime Silk Road, designed to tie China to Southeast Asia, Africa, and Europe [17]. The initiative has entailed heavy investments in large-scale infrastructure projects, including the creation of Eurasian land bridges and maritime transport corridors. Having grown since to encompass 150 countries [18] as well as new elements like the digital, space and health silk roads, the BRI has become “part of China’s geopolitical code of creating connectivity and cooperation, with an additional aim, or at least implication, of reducing US hegemony, especially US influence in Asia” [19]. In terms of scale, Beijing has invested $354.25 billion and spent $531.11 billion in construction projects through the BRI rubric [20]. Despite concerns over sustainability and quality of the projects [21], as well as so-called “debt trap diplomacy” [17], the BRI “has undeniably helped Beijing to deepen economic and political ties with emerging nations across Asia and Africa” [22:82].

Although Donald Trump’s trade war with China and spats with allies garnered the most headlines and at times hampered the administration’s initiatives, contrary to conventional wisdom his team actually sought to strengthen US alliance networks in the Indo-Pacific in response to Beijing’s multi-pronged challenge [23, 24]. The administration’s approach to the Indo-Pacific was laid out in various policy speeches by key officials from 2017 to 2019 [25] and culminated in the launch of the Indo-Pacific strategy in November 2019. Building on an idea initiated by Japan, the USA adopted a vision for a Free and Open Indo-Pacific (FOIP). The strategy was premised on “respect for sovereignty”, “peaceful resolution of disputes”, “free, fair and reciprocal trade based on open investment, transparent agreements and connectivity” as well as “adherence to international law” [26:6].

In other words, at the centre of the FOIP construct is safeguarding a rules-based order in the Indo-Pacific region [27], and in this sense, it presents a continuation of the Obama administration’s attempts to “pivot” or “rebalance” to Asia. In fact, also the term “Indo-Pacific”, joining together the two great oceans, had gained purchase during Obama’s term at both the Pentagon and the Department of State [28]. Still, by adopting FOIP as a “whole-of-government approach” [28], the Trump administration ultimately mainstreamed a new conceptual framing in US foreign and security policy parlance [24]. Even the Pacific Command (USPACOM) was renamed the *Indo*-Pacific Command (USINDOPACOM). Unlike its predecessors, Trump’s team was also explicit that the purpose of its approach was to contain and potentially confront China [29:2].

The Biden administration has continued in this vein, pledging to “shape the strategic environment around Beijing to advance our vision for an open, inclusive international system” [30]. Under the banner of “invest, align, compete”, the administration seeks to rebuild the domestic foundations of American power, re-engage with US allies in the region and globally, and face off against China in technological, economic, and military domains [30]. To these ends, the Biden administration unveiled its new Indo-Pacific strategy on 11 February 2022. It defines the Indo-Pacific as “stretching from our Pacific coastline to the Indian Ocean” [31:4],^2^ and lays out five objectives, namely “advance a free and open Indo-Pacific; build *connections* within and beyond the region; drive regional prosperity; bolster Indo-Pacific security; and build regional resilience to transnational threats” [31: 7]. For the Biden administration, then, FOIP entails creating a space “where governments can make their own sovereign choices, consistent with their obligations under international law, and where seas, skies, and other shared domains are lawfully governed” [31:8]. In the Indo-Pacific action plan, ten core areas of focus are identified: “Drive new resources to the Indo-Pacific”; “lead an Indo-Pacific Economic Framework [IPEF]”; “reinforce deterrence”; “strengthen an empowered and unified ASEAN”; “support India’s continued rise and regional leadership”; “deliver on the Quad”; “Expand U.S.-Japan-ROK cooperation”; “partner to build resilience in The Pacific Islands”; “support good governance and accountability”; and “support open, resilient, secure, and trustworthy technologies” [31: 15–17]. Notably, after a Trumpian aberration, Biden has again brought democracy and human rights centre stage in US foreign policy discourse. In fact, the President sees a world defined increasingly by a struggle between democracy and autocracy [33].

The emphasis on democracy—in particular the need for democratic, like-minded countries to promote shared values including the rule of law—has been strongly supported by Japan. It is also the core idea behind the Indo-Pacific concept, of which Tokyo has been a key driver. For Japan, the Indo-Pacific denotes a strategic term for a wider region that combines two continents (Asia and Africa) and two oceans (the Pacific and the Indian Ocean) [34]. While the Indo-Pacific moniker itself is new, it needs to be kept in mind that as a geopolitical and single strategic system, the region is much older and had arguably been in place for close to two centuries until the start of the Cold War era [35]. The return of the Indo-Pacific as a strategic concept is often traced back to the seminal speech “Confluence of Two Seas” by former Prime Minister Shinzō Abe in August 2007 at the Indian parliament. This was at a time when Japan was trying to promote its interests and values based on the idea of an “Arc of Freedom and Prosperity” (2006) along the outer rim of the Eurasian continent. The “Broader Asia” that Tokyo envisaged implies a maritime region that bestows a key strategic role to India as a regional power. After Abe returned to office in 2012, he strongly aimed to advance the idea of India and Japan coming together as guardians of navigational freedom in the Indian and Pacific Oceans. Abe’s notion of a “Democratic Security Diamond” (2012) formed by Australia, India, Japan, and the USA to safeguard the maritime commons can be seen as a direct precursor of the FOIP construct.

In 2016, the Indo-Pacific idea came to the fore in the context of a collective rhetorical commitment to a region that is “stable”, “open”, and “free”, and is connected to fundamental values including freedom, democracy, and human rights as well as strategic interests, particularly freedom of the sea lanes—ergo the *Free* and *Open* Indo-Pacific. The “strategy” was born after China’s BRI received traction in Southeast Asia in particular, and following the 2016 South China Sea arbitration ruling (Philippines v. China), which dismissed China’s claims of historic rights over the maritime areas [36: 55–56]. Most observers agree that FOIP is implicitly an attempt to counterbalance China, even though Japan has emphasized its vision is inclusive. For Japan, FOIP seeks to promote three core ideas: (1) rule of law, freedom of navigation and free trade; (2) economic prosperity (including through improvement of connectivity); and (3) peace and stability through maritime security (including through maritime law-enforcement capacity-building).

In terms of policy practice, Japan’s FOIP has focussed on five issue areas: (1) promoting public diplomacy on maritime order and the FOIP vision, (2) rule-making to expand free and fair economic partnership, (3) expanding connectivity, (4) enhancing capacity-building, and (5) ensuring maritime security. FOIP for Japan has normative, political, defense-related, economic, and security implications [37]. Normatively, FOIP is about identity-building, creating a new, strategic regional definition, and shaping the region’s rules, norms, and expectations. Politically, it seeks to emphasize selected elements of the US hegemonic liberal order, without Tokyo being perceived as a proxy for American power. In the sphere of defense, in the past decade or so Tokyo has increased and recalibrated its defense budget. In order to reinforce the alliance with the USA, Japan has approved the creation of a US-style National Security Council, passed a Secrecy Bill, and reversed a self-imposed ban on exercising the right to collective self-defense. Most recently, in December 2022, Japan unveiled a new National Security Strategy (NSS). Tokyo declared its intention to double the current defense spending during the 5-year-period up to 2027, bringing it within the range of 2% of GDP, as well as to acquire counter-strike capabilities. FOIP is complementary to Tokyo’s national defense posture—it entails balancing against China only implicitly, whereas in the wider defense policy, this is more explicit [37: 10–11].

Economically, FOIP allows Japan to link connectivity with “quality infrastructure investment”, and, along with this, a greater use of Official Development Assistance (ODA) for strategic purposes, including in Africa. Tokyo has been re-emphasizing economic statecraft and a geo-economic agenda, i.e., the use of trade and economic means in order to achieve security-related and geopolitical goals. “Securitized” ODA is also key in the security-related dimension, where Japan aims to build up stronger regional resilience and deter coercion. It is Tokyo’s ambition, through its support for maritime security capacity-building, to better equip other states to defend their maritime spaces and uphold international law. As for security, FOIP also works at the level of fortifying alliances and strategic partnerships. After the USA’s adoption of FOIP, Japan has greatly welcomed Washington’s re-engagement in the region. The focus on the Japan-US alliance as the linchpin of Japanese foreign policy remains. However, Japan has poured more effort into pursuing increasingly multifaceted, strategic diplomatic relations with other countries and regions as part of a widening alignment policy, forging strategic partnerships with Australia, India, ASEAN countries, and Europe, as well as strengthening cooperation with NATO [38].

## Connectivity: a Concept in Search of Meaning

Great-power competition is increasingly unfolding over the establishment, maintenance, and severing of different *connections*. In his *Age of Unpeace*, Mark Leonard argues that “connectivity conflicts” should be viewed as “the core organizing principle of today’s world” [39: 16]. What is more, connectivity has become a ubiquitous term in the policy parlance of diverse international actors. This proliferation in its usage alone makes connectivity an essential concept to grapple with.

For present purposes, connectivity entails “[a]ll the ways in which states, organisations (commercial or else) and societies are connected to each other and interact across the globe” [40: 1, 41]. It is a metalogic of international ordering [2] that encompasses “both the physical flows of people and goods as well as information flows [… and] covers ‘hard’ infrastructures as well as ‘soft’ regulatory measures or socio-cultural ties” [40: 1]. In this vein, as Gaens, Sinkkonen, and Vogt posit in this special issue, connectivity covers different spheres: material infrastructures, the economy, institutional frameworks, the exchange of knowledge, socio-cultural exchange, and security [2]. Connectivity thus pervades all levels of analysis potentially relevant for the study of international politics, from the interstate and transnational all the way to local.

The definition of connectivity above leaves open two crucial questions: why actors connect and how the boons from connectivity are distributed. We perceive connectivity as a functional phenomenon, undergirded by intentional rationality on the part of “connecters” and “connectees”. Thus understood, connectivity can come about as a result of a search for profits or chiefly altruistic reasons, but in the real world the goals of actors likely fall somewhere in between these poles. In addition, connections can breed benefits for both, or be entirely exploitative, with, again, considerable variation across the space straddling these extremes.

As Gaens, Sinkkonen, and Vogt posit, connectivity can be further unpacked in terms of six *logics* [2]. Ideally, connectivity is an exercise in inclusion. At best, it functions in accordance with a *cooperative* logic, as emphasized by liberal theories of international politics, in that “peoples and governments have a deep common interest” in cooperating, while “trade and exchange have a modernizing and civilizing effect” [8: 63]. In such a world, “connectivity has replaced division as the new paradigm of global organization” [42, xvi]. Connections, from this standpoint, breed order. The diffusion of connections around the world also creates space for convergence, and the *copying* of best practices from others, which, again, enhances further connectivity. Finally, inclusive connective networks also provide space for actors to broaden their range of connections, to *cushion* against potential risks in a particular connectivity sphere by connecting with many providers at the same time. This is akin to hedging in IR literature [11, 43].

However, connectivity can also be exclusionary in nature, and even drive dynamics of disorder in the process. Connectivity is both a potential site and means of *contestation*. Such competition can, of course, unfold in a rule-governed manner, or, in the case of free markets, be an essential mechanism for driving out inefficient producers of connectivity. However, recent analyses of “weaponized interdependence” [44: 45] or “connectivity wars” [45] illustrate how states can use their privileged position in a connected network to either *contain* other actors, hindering their connectivity or excluding them from connecting altogether, or *coerce* others, “through infliction of pain or damage—or the withdrawal of something valued (or the threat thereof)” [46: 58] to engage in (dis)connections they would otherwise not undertake.

This short exploration of the logics of connectivity, as laid out in more depth by Gaens, Sinkkonen, and Vogt, underlines how connectivity can be constitutive of a state’s power, and more deeply still, global order [2]. In this vein, connectivity directs our attention to relationships between actors straddling different levels of analysis and places such relationships at the centre of analyses on global power and even hegemony [47]. This renders connectivity and its logics vital for understanding the parameters and future trajectories of great-power competition and prospects for order.

As such, connectivity has particular relevance in the context of the Indo-Pacific. Per Rory Medcalf, the Indo-Pacific is a “super-region”,^3^ which presents “an *expression of global connectivity*: the main highway for commerce and energy between Asia, Africa, Europe, Oceania, and the Americas […] the most globally *connected* of regions” [49: 91]. The region is central to the connectivity project par excellence, namely China’s BRI, to which the connectivity forays of other regional or global powers, including the USA and Japan, are implicit or explicit reactions. Moreover, as we endeavour to illustrate through our empirical examples, connections bring concretia to the entire Indo-Pacific construct, which has been criticized in the literature for being either an illusory notion or merely a new geostrategic imaginary constructed to contain China [cf. 50, 51–53]. Connectivity, therefore, is central to the processes of institutionalisation that have been raised in debates about the Indo-Pacific as necessary for making this superregion “real” in the coming years [52, 54, 55]. We argue that inclusivity or exclusivity of the established connections will thus ultimately determine whether convergence around the construct creates stability and peace (order *constitution*), or competition and even potential conflict (order *fragmentation* or *corrosion*).

## The Connectivity Policy of the United States: Rhetoric Meets Reality

While connectivity per se is a relatively novel term in US foreign policy parlance, the USA has been a guarantor of global connectivity for decades, if not centuries. As Christopher Layne argues, commitment to the economic and political “Open Doors”—to “maintaining an open international system” and “spreading democracy and liberalism abroad” are long-term constants of US grand strategy [56: 30]. Moreover, to sustain its hegemonic role in the post-World War II liberal international order, the USA has provided global public goods as part of a “hegemonic bargain” with its allies and partners (although others, including China, have benefited as well) [57, 58]. Through its military and economic primacy, the US has “commanded the commons” [59], keeping key global arteries like sea lanes or the global financial system open, hence ensuring unimpeded “flows” of tangibles and intangibles [60]; in essence, then, the USA has ensured and enabled connectivity for itself and others.

Yet, in the recent past, the USA has not spent large proportions of its aid on infrastructure projects, instead spending vast amounts of its largely grant-based assistance on “soft” connectivity, including health, humanitarian assistance and governance reform [61: 6]. The USA has prioritized infrastructure projects in countries that fill good governance criteria through the Millennium Challenge Corporation (MCC) and has sought to stimulate private sector investment through bodies like the US International Development Finance Corporation (DFC) and the International Transaction Advisory Network (ITAN) [61: 16−17] 

In terms of total ODA, the USA ranks first among the Development Assistance Committee (DAC) countries at over $35.47 billion.﻿^4^ However, the regional distribution of ODA has not so far reflected the USA’s increased emphasis on the Indo-Pacific. In Fiscal Year (FY) 2020, for instance, none of the top-10 recipients of US ODA were in the region [62]. In fact, for FY 2020, the total tally of economic foreign assistance obligations for East Asia and Oceania was less than $1.5 billion, and even with the inclusion of India, Pakistan, Nepal, Sri Lanka, and Bangladesh, the figure does not exceed $2.5 billion – this amounts to roughly 7% of all US economic foreign assistance obligations for the year.^5^ The potential silver lining is that US foreign direct investment (FDI) in the region for 2020 was touted at $969 billion [63], constituting a resource to be tapped into for future connectivity endeavours. While the development/security nexus has been ever more prevalent in US foreign assistance in the post 9/11 era [61:1–3], recent discourse links it to strategic competition with Russia and China, and the BRI in particular [64:3–4].

Already the Obama administration’s pivot/rebalance to Asia included an emphasis on connectivity in manifold spheres. To illustrate, the “New Silk Road” plan was an early attempt to enhance connections in Greater Central Asia and link Afghanistan to the Indian Ocean trade routes via India, the US-ASEAN Connect was introduced to create more strategic focus in US economic engagement vis-à-vis the region [65], and the Global Procurement Initiative (GPI) was unveiled to aid partner countries in establishing “effective public procurement systems” for infrastructure development [66]. The USA also launched the Lower Mekong Initiative in July 2009 with Cambodia, Laos, Thailand, Vietnam, and (later) Myanmar to “enhance cooperation in the areas of environment, health, education, and infrastructure development” [67]. Most importantly, the Obama administration wanted the USA and its partners, not China, to set the rules and standards of regional trade for the twenty-first century. The Trans-Pacific Partnership (TPP), a headline free-trade initiative encompassing twelve Pacific rim economies, was supposed to achieve this goal [68].

The Trump administration abandoned this trade plank by pulling the USA out of the TPP, which was later rebranded the CPTPP (Comprehensive and Progressive Agreement for Trans-Pacific Partnership) by the remaining members. Yet, the USA’s stance on China hardened noticeably. Per the 2017 NSS, “China seeks to displace the United States in the Indo-Pacific […] and reorder the region in its favor” [69:25]. Of course, a strong domestic-political element underpinned Trump’s China policy, centering around China’s trade surplus vis-à-vis the USA and the loss of American manufacturing jobs [70]. During his tenure, the President instituted a full-blown trade war against Beijing, keeping the trade restrictions in place even after the two agreed on a “Phase One” trade deal in January 2020. In the military domain, the administration also upped the frequency of Freedom of Navigation Operations (FONOPs) in the South China Sea, with the aim of upholding international law and contesting what it regarded as China’s “excessive maritime claims” [71].

The USA also concocted an alphabet soup of initiatives to go with its FOIP approach and challenge what the USA deemed China’s “investments in the developing world to expand influence and gain competitive advantages against the United States” [69: 38]. In July 2018, Secretary of State Mike Pompeo announced a rather modest “$113 million in new U.S. initiatives to support foundational areas of the future: digital economy, energy, and infrastructure” [72]—to effectively make the region more connectable in keeping with the FOIP principles and do so in a manner that would benefit US security interests, harness the resources of the private sector and benefit US firms and American workers. Initiatives included a Digital Connectivity and Cybersecurity Partnership (DCCP) [73], Asia EDGE (Enhancing Development and Growth through Energy) [74], as well as an Infrastructure Transaction and Assistance Network (ITAN) [72]. Later that year, a $10 million US-ASEAN Smart Cities Partnership (USASCP) [75] and a $9 million technical assistance program called the US-Support for Economic Growth in Asia (US-SEGA) [76] were added to the mix. Building on the Lower Mekong Initiative—which had delivered a total of $3.5 billion to the area between 2009 and 2020—the USA, in 2020, announced the Mekong-US Partnership with new investments, particularly in the field of energy [77]. The Trump administration also placed increased emphasis on the Pacific Islands with its “Pacific Pledge”, $100 million on “economic and environmental resilience, maritime security, and good governance” unveiled in 2019 and upped to $200 million in 2020 [78].

Alongside such initiatives, the administration sought deepened collaboration with regional partners. Together with Japan, Australia and India, the USA resurrected the Quadrilateral Security Dialogue (Quad), a grouping originally in place during 2007–2008. The first of a series of ministerial-level meetings took place at the November 2017 ASEAN Summit. In 2017, a bilateral MOU was signed between the US Overseas Private Investment Corporation (OPIC) and the Japan Bank for International Cooperation (JBIC), with the aim of “support[ing] potential projects in […] sectors, such as infrastructure, energy and natural resources” [79]. The following year, Australia was brought along in a trilateral memorandum of understanding “to support projects like 21st Century infrastructure, energy, and telecommunications to increase regional connectivity” [80]. The USA and Japan also launched the Japan-US-Mekong Power Partnership (JUMPP) focussed on energy sector reform [81]. Finally, at the 35th ASEAN Summit in November 2019, the Trump team launched the Blue Dot Network (BDN)—again in cooperation with Japan and Australia—envisaging a certification mechanism “to promote quality infrastructure investment” that complies with inclusivity, transparency, and environmental and sustainability standards [82].

Congressional activism was also pronounced during Trump’s tenure. In October 2018, Congress passed the Better Utilization of Investments Leading to Development (BUILD) Act, which reorganized US development financing. DFC, a new body, took on the tasks of OPIC and charge of the USAID Development Credit Authority (DCA) with a threefold mandate comprising development impact, US foreign policy goals, and returns for US taxpayers. Unlike OPIC previously, the DFC can make equity investments and has an increased investment cap ($60 billion as opposed to $29 billion previously) [83: 84, 84]. In December 2018, Congress signalled further support for US engagement in the Indo-Pacific with The Asia Reassurance Initiative Act (ARIA) [85]. Containing an explicit focus on the role of alliances, partnerships and regional institutions, the ARIA was also a rebuke of the President’s tendency to publicly undermine such cooperative forays [86].

The Biden administration came into office resolved to rejuvenate US global leadership and reunite the country by addressing its manifold domestic challenges, including the Covid-19 pandemic, economic inequality and crumbling infrastructure. Even the administration’s headline initiative in meeting the $40 trillion infrastructure deficit in low- and middle-income countries was dubbed Build Back Better World (B3W), a nod to Biden’s push for the Build Back Better (BBB) Act at home. Announced at the G7 meeting in June 2021, the B3W initiative envisaged “four areas of focus—climate, health and health security, digital technology, and gender equity and equality” [87]. Moreover, it was framed as a “values-driven, high-standard, and transparent infrastructure partnership led by major democracies”, a thinly veiled reference to the criticisms raised towards the BRI [87].

The June 2022 G7 Summit relaunched the B3W as the Partnership for Global Infrastructure and Investment (PGII). The rubric pledges $600 billion to infrastructure projects over the next 5 years, and $200 billion of this pot will be provided by the USA. More focus on “hard infrastructure” has been added to assuage criticism that the B3W did not sufficiently address such needs of low- and middle-income countries. This makes PGII a potentially more potent competitor for the BRI [88]. Among the projects initially announced is “a 1000-mile submarine telecommunications cable that will connect Singapore to France through Egypt and the Horn of Africa” [89]. The inclusion of “energy security” and “digital connectivity” take account of the changing international environment after Russia launched its war of aggression on Ukraine on 24 February 2022 [90, 91].

Biden’s Indo-Pacific strategy departs from the Trump administration’s document by placing even more emphasis on the role of allies and partners as well as introducing the Indo-Pacific Economic Framework (IPEF), trying to rectify the omission of a multilateral trade component in the previous administration’s approach [92]. Unveiled during President Biden’s Japan visit on 23 May 2022, the framework encompasses twelve regional states, aims to entrench “high-standard commitments,” and is composed of four pillars: “connected economy”, “resilient economy”, “clean economy”, and “fair economy” [63].

The first years of the Biden era have also been marked by a flurry of other multilateral and minilateral initiatives. Tying “ASEAN centrality” to its FOIP construct at the virtual US-ASEAN summit in October 2021, the administration pledged a $102 million investment in health, climate, economic growth, and people-to-people initiatives [93]. Following up on these pledges, at the 2022 summit in Washington D.C. on 12–13 May, the USA unveiled “over $150 million” worth of initiatives on climate (including $40 million through the Southeast Asia Smart Power Program), sustainable development, inclusive prosperity, education ($70 million), maritime cooperation ($60 million), and health [94].

Notably, the Quad has been further upgraded to a leader-level venture, with four Quad leaders’ summits held in 2021 and 2022 (two of them in-person). The FOIP construct remains the headline commitment of the grouping, and the partners have pledged to lead on high-quality infrastructure in the auspices of “a new Quad infrastructure partnership”. The infrastructure plank complements a broad set of priorities, including coordination on the pandemic, climate, and technology [95, 96]. At the May 2022 Tokyo Summit, the Quad countries pledged $50 billion in infrastructure assistance across the Indo-Pacific for the coming 5 years, while also setting up a Quad Infrastructure Coordination Group [97].

In a reflection of the challenges posed by the Covid-19 pandemic for the Indo-Pacific region, and in reaction to China’s “vaccine diplomacy” [98], the virtual Quad leaders’ summit in March 2021 pledged “equitable vaccine access for the Indo-Pacific, with close coordination with multilateral organizations including the World Health Organization and COVAX [COVID-19 Vaccines Global Access]” as well as a “vaccine expert working group” to spearhead this process [99]. The Quad’s 1 billion by the end of 2022 vaccine pledge was upped to 1.2 billion at the in-person September 2021 meeting. The idea was to manufacture the vaccines in India, the USA would provide the financing, while Japan would aid in paying, e.g., cold-chains. Australia, in turn, would take on distribution tasks in Southeast Asia and the Pacific [95]. The May 2022 Tokyo Summit touted the Quad’s achievements: it had provided 40% or $5.2 billion of the funding for COVAX’s Advance Market Mechanism, and delivered, at the time, 265 million doses to the Indo-Pacific region [97, 100]. More generally, the Quad is a vital component in the Biden administration’s resolve to keep Beijing from rewriting “the rules and norms that have benefitted the Indo-Pacific and the world” [31:5]. The group’s focus on the FOIP notion also fits with Biden’s free world agenda, headlined by the inaugural Summit for Democracy held on 9–10 December 2021.

The plethora of connectivity initiatives the USA has set up in recent years, on its own and in conjunction with its partners, are undeniably a reflection of the urgency with which Washington views China’s growing influence in the region. However, the US approach suffers from at least four interrelated problems.

First, the role of domestic politics looms large. As Choong has argued, Trump’s FOIP approach—in contrast to those of US partners Australia, India, and Japan—included “more of a domestic slant […] focused less on encouraging Beijing to adhere to FOIP principles, and more on pressurising China to reduce its trade surplus with the U.S., abandon its ‘made in China 2025’ technology initiative and eliminating tariffs” [101:420]. This situation has only partly changed in the Biden era. In particular, the administration’s commitment to a “Foreign Policy for the Middle Class” has entailed a cautious trade policy [102]. While the USA has sold the IPEF as a template for regional economic leadership in the twenty-first century, lukewarm reception by regional partners suggests that the lack of improved access to US markets in the proposal cannot be offset by a focus on issues like supply chains, climate change and infrastructure [103].

Second, despite the high-flying pledges, the US headline initiatives for contesting the BRI have so far appeared insufficient [104]. Consider the BDN: with the change of administration, further development of the framework has taken place under the auspices of a multi-stakeholder OECD-led Informal Executive Consultation Group, which has so far met three times and produced an “evidence-based proposal for a credible, efficient, transparent and sustainable certification framework that can provide a basis for operationalising”. The BDN is off to a slow start and is just now in the process of carrying out pilot projects [83: 85, 105]. Critics also point out that the Biden administration’s initiatives for US-ASEAN cooperation appear scattershot, missing an overarching narrative regarding the future direction of the relationship as well as sufficient funding [106]. As of June 2022, the B3W had made little progress to speak of: one analysis placed the value of projects kicked off under the banner at a meagre $6 million [107]. While the PGII might yet prove an improvement upon the stalled initiative, according to one observer, it looks like “a gamble” on an untested concept, namely that governments “can mitigate enough risk that private capital will feel more comfortable investing in identified projects” [88]. All this speaks to a “say-do gap” in Biden’s Indo-Pacific strategy, namely “[i]t reads more like a wish list than a strategy, which would require actual trade-offs” [108]. A further challenge for the PGII is less-than-enthusiastic reception in key partner countries, especially India [109].

Even the few successes remain qualified. In the case of Covid-19 vaccines, for instance, the USA has placed much focus on the Indo-Pacific, as the top-5 recipients of US vaccines (Bangladesh, Pakistan, Vietnam, Indonesia, and the Philippines) are all located in the region [110]. The Quad vaccine partnership, however, remains well short of its 1.2 billion doses by the end of 2022 target, and its provision of vaccines to the region falls far behind Western bilateral donations as well as China’s vaccine sales [111, 112]. The minilateral cooperation with US allies in the region has brought only limited success: the Trilateral Partnership with Japan and Australia has produced one substantive project, a fiber optic cable that links Palau with the Indo-Pacific [113]. Possible future templates for sustainable infrastructure development within the G7 rubric are the Just Energy Transition Partnerships (JETP), piloted in South Africa and currently under discussion with Indonesia, India, and Vietnam [89, 114], yet these forays, too, remain in their infancy.

Third, there is a broader debate about how sensible it is for the USA and its partners to contend directly with the BRI. While some have called for the USA to stick to its strengths in “soft” connectivity arenas like digital trade, health, and education [115, 116], and focus on supporting MDBs’ “hard” connectivity projects [107, 117], others have suggested doubling down on funding for infrastructure development [118]. Hence, the novel DFC, for instance, has faced criticism from many sides: for channeling insufficient resources to countries where China has had a strong foothold through the BRI, for not competing directly with China in hard infrastructure [119, 120], as well as for as skewing focus away from development [91].

Fourth, the Biden administration has framed connectivity as a vital front in an era that, per the President, is marked by “a battle between the utility of democracies in the twenty-first century and autocracies” [121, see also 33]. However, it is possible that the strong values-based component to the US Indo-Pacific strategy might strain relations with those very states—like the Philippines, Singapore, Thailand, and Vietnam—that the USA wants to partner with in the region but that are hardly posterchildren for democracy [122]. More broadly, it is evident that states in the region prefer not to make a choice between the USA and China, instead maintaining their agency and ability to “cushion” between the two great powers [123]. Challenges to US connectivity projects can thus flow from the domestic arenas of potential partners. In February 2022, for instance, a $500 million MCC grant to Nepal for improving the country’s road and electricity infrastructure sparked widespread protests, motivated by local fears that entanglement with the USA would jeopardize relations with China [124].

## Japan’s Policy: a Connectivity Superpower Struggling to Cooperate

Japan’s connectivity policy is strongly linked with its development cooperation, which itself has traditionally focussed on infrastructure development. This is rooted in the country’s historical experiences, as Japan focussed its post-war development on rebuilding its economy. Japanese ODA has therefore centred principally on infrastructure development and capacity-building in order to help recipient countries develop a functioning market economy and increase self-reliance. Also today, Japan’s development cooperation focusses on infrastructure and connectivity, in particular economic infrastructure. Transport and storage, communications, electricity, banking and financial services, and business support comprise 52.1% of Japan’s total bilateral ODA (as opposed to 4.9% for the USA), and amount to $7.7 billion in 2019. This compares to 13.7% of bilateral ODA dedicated to social infrastructure such as education, health, population policies, water and sanitation, and government and civil society (as opposed to 41.5% for the USA) [125:173]. Tokyo’s key tools have been low-interest, long-term loans instead of grants (68% of the total in 2020 [126]), and widespread use of public–private partnerships, primarily with Japanese companies. A key instrument here is the Japan International Cooperation Agency’s (JICA) Private-Sector Investment Finance tool, which provides loan aid to private corporations engaging in infrastructure development to assist them in local development projects.

Being a regional power, Japan has typically focussed its infrastructure development policy on Asia, in particular Southeast Asia, grounded in solid economic, diplomatic, and geographical links. At present Japan still invests more than China in ASEAN economies such as Indonesia, Malaysia, Philippines, Thailand, and Vietnam. It has $259 billion invested in unfinished projects in these countries, compared to $157 billion for China [127]. According to another source [128], public and private financing from Japan stands at $330 billion across ASEAN, with Chinese investment amounting to $100 billion. Tokyo is also investing robustly in connectivity and development projects in Indonesia, Vietnam, and the Philippines, often through ODA loans at 0.01% interest rate [128].

The Partnership for Quality Infrastructure (PQI), launched in 2015 and budgeted at $110 billion (and in 2016 raised to $200 billion), is a key policy tool to promote connectivity. The emphasis on quality, including connotations of economic efficiency, safety, resilience, environmental and social sustainability, and contributions to local society and economy, denotes a clear attempt to set Japan’s policy off against China’s and counterbalance the BRI [129:14]. The PQI is based on four pillars: (1) expand ODA loans for Asia’s infrastructure and mobilize private funding; (2) strengthen collaboration with the ADB including by promoting PPP (public–private partnerships) for infrastructure investment by utilizing JICA’s Private Sector Investment Finance; (3) increase funding for high-risk infrastructure investments through the Japan Bank for International Cooperation (JBIC) and a newly founded Japan Overseas Infrastructure Investment Corporation (JOIN); and (4) promote “quality infrastructure investment” as an international standard [130].

In recent years, Japan has expanded its geographic outlook as an outcome of the Abe administration’s FOIP vision, aiming to connect Asia and Africa and promote sustainable growth in both regions. In particular, and as a result of a growing awareness of the importance of connectivity in regional power dynamics, Japan has been broadening its focus under the FOIP to include Africa, South Asia, and the Pacific Islands, and to complement hard (physical) connectivity with soft connectivity (people-to-people contacts and capacity-building). Examples include the Bay of Bengal Industrial Growth Belt (BIG-B) in South Asia and the East Africa Northern Corridor. At the most recent Tokyo International Conference on African Development (TICAD8, August 2022), Japan pledged public and private investments of $30 billion over the next 3 years, including a green growth initiative, an investment fund for startups, and support for human resource and private sector development.

What is new in Japan’s connectivity and development strategy is the wider quest for strategic partnerships with third countries and regions, primarily as a result of increasing competition with China, but more recently also because of Russia’s aggression in Ukraine. As for cooperation with the USA, Japan is the only one of the US’s regional partners to have fully endorsed the Biden administration’s IPEF, not least to strengthen diversification away from China. Tokyo believes IPEF will not only strengthen the US-Japan economic partnership, but also hopes it can be a stepping stone for the USA to return to the CPTPP [131]. The 14 members of IPEF agreed to start formal negotiations for a rules-based economic order in the region in terms of trade, supply chain resilience, clean energy, and fair economy. Japan is also part of the PGII and has pledged to contribute over $65 billion over the next 5 years in government and private sector investments. Further recent cooperative endeavours include the Japan-US Clean Energy Partnership (JUCEP) to support decarbonization efforts in the Indo-Pacific, and the US-Japan Global Digital Connectivity Partnership (GDCP) to promote international data flow rules. Lastly, the above-mentioned Palau Cable 2 (PC2) optical submarine cable construction project supported by Japan, the USA and Australia comprises insurance underwriting by Japan’s NEXI (Nippon Export and Investment Insurance) and financing by JBIC.

This cursory overview of Japan’s connectivity policy yields five key takeaways. First, Japan’s connectivity policy is closely linked to its domestic economic and security agenda. Japan has always strongly emphasized its own national interest in allocating ODA, and has explicitly linked investments, in particular in economic infrastructure, with development cooperation in order to support its own export sector and buttress its economic and foreign policy interests. This has been the case especially in Southeast Asia. Ever since the 1970s, Japan has employed infrastructure development as a tool to help Japanese companies access local markets. In recent years, development policy has become increasingly securitized, and ODA has been used to support foreign military forces for “non-military purposes”, for example. ODA has, therefore, also been a central component of Japan’s FOIP vision. This has led to a perception of Japan’s ODA aiming in the first place to balance the growing power of China, even if, unlike the more directly confrontational stance by the USA, Japan has mainly tried to strengthen its economy by improving connectivity and cementing economic partnerships in the Indo-Pacific [132:7−11].

Second, Japan has undeniably been a “connectivity superpower” long before the concept of connectivity came in vogue, and long before China launched its BRI. As the largest ODA provider in Asia with a heavy focus on economic infrastructure investment, Japan’s connectivity-related involvement in Southeast Asia is still larger than China’s. Based on the Japan-ASEAN Connectivity Initiative, Japan is further strengthening its involvement in the region, through the development of a Land Corridor and a Sea and Air Corridor. The former comprises an East–West Corridor from Vietnam through Laos and Thailand to Myanmar, and a Southern Corridor from Vietnam to Cambodia, Thailand, and Myanmar. The latter involves projects in Cambodia, Myanmar, and Indonesia. While the focus is on physical connectivity, the projects are buttressed by technical cooperation (institutional and people-to-people connectivity). All in all, Tokyo is involved through ODA loans in numerous so-called ASEAN 47 network ports and in 33 flagship projects, amounting to a total of ¥2 trillion ($14 billion) [133].

Third, in terms of the say/do gap already noted in the case of the USA, Japan has been less successful so far in implementing tangible cooperative endeavors with third-country partners. In 2017, Japan signed a partnership agreement, labeled the Asia Africa Growth Corridor (AAGC), with India, focussing on economy, technology, and infrastructure development in the Indo-Pacific and Africa, and building on Japan’s PQI and India’s “Act East” policy. The policy attracted a lot of attention and was followed by high expectations. However, on the ground, no progress has been made, to the point that after five years the project has been called a non-starter [134]. Furthermore, in September 2019, the EU and Japan concluded a Partnership on Sustainable Connectivity and Quality Infrastructure. A long list of EU-Japan synergies, complementarities, and tangible cooperation in the field of development cooperation in Southeast Asia, the Pacific region, Central Asia, and Africa was compiled in 2021 [135], but three years after the start of the partnership, its implementation remains vastly underwhelming.

Fourth, competition with China takes on a central role in Japan’s connectivity policy. For example, contending with China is a strong driver for increased investment in Africa under FOIP. For Japan, the development of the Mombasa Port in Kenya is pivotal. Aiming to prevent China from gaining influence over the port, Japan invested in an SEZ (Special Economic Zone), provided 17 patrol boats to Kenya and offered capacity-building training to local coastal guard officers. Furthermore, Tokyo has aimed to use multilateral channels including TICAD, as opposed to China’s bilateral approach [136:16−17]. In Southeast Asia, at least until the military coup of 2021, Myanmar was key in Japan’s goal to counterbalance China’s BRI. Since 2015, Japan has invested heavily in the Thilawa Special Economic Zone, key in the East–West Economic Corridor. The nearby Thilawa Port is vital as a gateway to the Indian Ocean for Japanese manufacturing companies. Japan also aimed to invest in the Dawei SEZ, including a deep-sea port vital for the development of the Mekong Southern Economic Corridor (SEC). In terms of security, and restricted by its constitution, Japan has widely been providing maritime law enforcement assets to Djibouti, Indonesia, Malaysia, the Philippines, Sri Lanka, and Vietnam [36:68]. Unlike the USA, Japan, with its focus on infrastructure, is a direct contender to China in regions such as South East Asia. In other regions such as Africa, cooperation with partners such as the USA, the EU, and India is probably more essential.

Fifth, when it comes to values-based diplomacy in relations with third countries, Japan has been employing the rhetoric of democracy and human rights in order to rally partners behind its FOIP cause, but in practice, Tokyo has taken a very pragmatic stance. Japan’s FOIP has evolved over time, and, in particular, the emphasis on basic values such as democracy and human rights has been toned down strongly in order to avoid criticism of meddling in the internal affairs of partner countries. Importantly, Japan changed FOIP from a “strategy” into a “vision” in 2018, in response to criticism from ASEAN countries that the former concept strongly denotes a regional major-power competition with China [36:65].

## Logics of Connectivity in the Indo-Pacific

Japan has a long-standing history of postwar development cooperation that predates its current connectivity efforts through the PQI. Japan therefore has abundant expertise in usefully combining collaboration with multilateral development banks, employing domestic financial institutions, utilizing ODA, and getting the private sector on board (including through investment guarantees). The US ODA contribution is massive, but it has a much less illustrious record in the realm of hard infrastructure development. While the country undertook large-scale investment in infrastructure development as a result of the wars in Afghanistan and Iraq, the USA has spent meagre resources on infrastructure development—to illustrate, the sum total of spending listed under infrastructure in US foreign assistance for 2019 was $943 million of the over $48 billion overall pot [61:5], reflecting US reliance on the private sector to fund such projects. Moreover, critics point out that the USA’s own crumbling infrastructure hardly makes it a credible player in this domain [114]—although this could ultimately change with the $1.2 trillion Bipartisan Infrastructure Law, passed in November 2021.

What do the cases of the USA and Japan tell us in terms of the logics of connectivity as outlined in the theory article by Gaens, Sinkkonen, and Vogt [2] in this special issue? First, both the USA and Japan evidently view connectivity as an indispensable tool to promote economic growth and prosperity, not least in light of the global need for infrastructure investment. Before the Covid-19 pandemic, the Asian Development Bank estimated that the infrastructure gap in the Asia Pacific amounted to $1.7 trillion a year [137]. Narrowing this gap is essential in order to maintain growth, eradicate poverty, and fight climate change, for example.

As such, it follows that opportunities abound for *cooperation* and joint infrastructure investment projects between key global actors. In effect, synergies between the ambitions of these actors have resulted in ambitious partnership plans in the sphere of cooperative connectivity in the Indo-Pacific region. As surveyed in this article, these include the Quad, B3W, PGII, BDN, AAGC, and the EU-Japan Connectivity Partnership. Cooperative connectivity is the ideal, and, all things being equal, it is also potentially constitutive of order more broadly.

However, Western-led cooperative connectivity plans often remain at the level of empty rhetoric, revealing a “capability/expectations gap” [138]. Eventually, a cycle of promises and rebranding without concrete results can lead to disillusionment on the ground. Multiple reasons and practical obstacles exist why cooperative strategies often do not exceed the level of an outline of synergies. These relate to insufficient funding, a lack of focus (e.g., whether to zero in on hard or soft connectivity), disregard of local actors, and insufficient leveraging of private sector investment. The last of these is essential. Indeed, what many Western connectivity plans, including the EUGG, the Quad partnership, and the B3W/PGII have in common is the aim to promote a long-term economic relationship rooted in private-sector investment, in other words, a focus on “mobilizing” or “leveraging” private finance [128]. However, private investors often shy away due to unpredictable political, regulatory and economic risks, in spite of risk-mitigating policies. This is even the case for countries such as Japan with a traditionally heavy emphasis on private sector involvement. Many companies shy away from investment in Africa for example, as political, security, economic, and regulatory risks are deemed too high. The case of Japanese firms’ unwillingness to invest in the port of Mombasa, Kenya, where Japan has a SEZ is a case in point [139]. In addition, an institutional ecosystem that allows for a speedy shift from the conceptual stage to a pipeline of bankable projects is lacking [140:11−12].

*Copying* ideally takes place through emulation and diffusion of norms and standards, for example. The USA adopting the originally Japanese FOIP concept is an obvious example, with profound repercussions for how the hitherto most powerful state in the international system thinks about the developing Indo-Pacific “super-region” [49]. In 2019, Japan was successful in international standard-setting when the G20 summit adopted Japan’s concept of quality infrastructure under the acronym PQII (Principles for Quality Infrastructure Investment) as a set of new principles for future projects. The template encompasses sustainable growth, economic efficiency, environmentalism, resilience, social awareness, and governance including openness and transparency of procurement.

In cases where viable options have been provided by the great-power contestants, *cushioning* opportunities have opened up for regional actors. For instance, Vietnam and the Philippines were able to expand their portfolios of Covid-19 vaccines as the USA and its Quad partners entered the vaccine diplomacy game in the Indo-Pacific, providing an alternative for China’s vaccine candidates [112]. However, in the long run, the possible failure of the West’s novel connectivity initiatives can also foreclose opportunities for smaller regional states and actors, and, in the process, decrease their agency if the BRI remains the only game in town.

Connectivity is inevitably linked with geopolitical agendas and often involves rivalry and *contestation*. Competition with China is key, and both the US and Japanese forays are defined, either implicitly or explicitly, as alternatives to the BRI. The Quad, although constituting a low-key and rather informal structure, as well as its driving vision, the FOIP, are constructed as counters to China’s policies. Yet, zero-sum competition *by definition* leads to zero-sum connectivity, where opposing great-power camps compete instead of focussing on their respective comparative advantages. Here, the debate on whether the USA and its allies *should* endeavour to compete directly with the BRI is salient. Even if the answer is yes, can Western value-based connectivity trump the BRI? An emphasis on quality inevitably involves slower implementation. In contrast, China’s BRI projects aim to achieve tangible results as quickly as possible, with less regard for sustainability. Connectivity thus risks turning into a race to the bottom in terms of quality measured by social and environmental sustainability [141:660–661]. As a result, even apparent successes can breed disappointment, deriving from overpromising. Arguably, channeling support through traditional MDBs whilst focussing on comparative advantages in “softer” fields, such as digital connectivity, could yield more tangible results [107, 117].

*Containment* dynamics are also present, most notably in military and economic spheres. US FONOPs in the South China Sea or “transits” through the Taiwan Strait are an obvious instance, aimed as they are at circumscribing China’s influence and upholding international legal principles in contested waters. Japan’s attempts to build up the maritime law enforcement capabilities of regional partners are (at least implicitly) meant to enhance their ability to resist China’s forays into disputed waters. The TPP/CPTPP, finally, was/is at its heart a project to contain China’s influence both economically and in terms of Beijing’s ability to set the normative parameters of the twenty-first century.

As for *coercion*, the social fallout of connectivity is clear, also in cases such as Japan’s infrastructure development projects through SEZs in places such as Myanmar and Laos. Forced relocations, absence of sufficient consultation with local stakeholders, lack of transparency and accountability in land acquisition, inadequate resettlement and compensation for lost land and livelihoods, the empty promise of jobs, along with social repercussions of environmental issues are just a few common issues [141, 142]. Furthermore, Japan has often been criticized for tying aid in connectivity projects to furthering its national economic objectives, linking Japanese companies to procurement contracts in recipient countries. Similarly, the USA has imposed sanctions under the Global Magnitsky Act against Cambodian authorities involved in leasing a port facility to China against American wishes [143].

## Conclusion: Policy Axioms and Implications for Regional Order

To conclude, the above analysis allows us to construct three policy axioms to inform a more sustainable US/Japan approach to connectivity in the region. First, there exists a clear need to *bring rhetoric and capabilities in line*. The USA’s manifest slowness in investing sufficiently in domestic infrastructure makes huge pledges for global connectivity seem even more incredible. Even for Japan, with its successful and long-standing infrastructure investments in Southeast Asia, for example, the achievements of initially promising cooperation agreements with India, the EU and the USA have been underwhelming.

Second, *Western actors (including the USA and Japan) need to be clear about the ultimate objective of their connectivity visions*. This entails answering the fundamental question whether the point of connectivity forays is to compete directly with China or to develop an approach that can complement Beijing’s initiatives. The commitment to FOIP is shared by many Western actors in some form, but disagreement exists over geographical scope, inclusive nature, and implementation. The USA has emphasized security and defense along with bi/minilateralism, whereas Japan has promoted free trade and continues to champion multilateralism alongside minilateral forays. There exists an obvious need to uncover focal issue areas and regions, outline synergies, coordinate policies, engage in standard-setting, and agree on joint funding. A suitable platform to set the baseline for this process would be the Asia-Europe Meeting (ASEM), a forum primarily for informal dialogue, in which connectivity has strongly come to the fore.

Finally, and relatedly, it is *essential to prioritize projects/spheres that are strategically important* and where the USA and Japan can best contribute and have a comparative advantage. JUMPP in the Mekong area is one current example. Here, the USA is not only playing to its strengths in enabling sustainable and renewable infrastructure development, but it is also partnering with Japan, and, as a by-product, building “soft power” by improving America’s desirability as a partner in the region [144]. Relatedly, Japan has a lot to offer in terms of expertise as for linking hard connectivity with “soft” infrastructure such as human resource development, management knowhow, normative standards, and principles in infrastructure investment [136:21]. In terms of offering a sustainable alternative to the BRI, capacity-building is essential in regions such as Africa, or in Southeast Asia, as in the context of JUMPP and MUSP mentioned above. Furthermore, Japan has vast expertise in bringing the private sector on board. The Japan-US Clean Energy Partnership (JUCEP) which aims to advance the deployment of renewable energy and decarbonization technologies through partnerships with the private sector will prove to be a fitting case study in this respect. The digital realm is another strategically important area where the USA and Japan can usefully cooperate, for example through the Global Digital Connectivity Partnership.

In terms of future prospects for regional order, the exposition above has illustrated the relative prominence of corrosive logics of connectivity (i.e., contestation in a zero-sum manner, containment, and coercion), which are evidently driven by key players’ preoccupation with great-power competition. To make matters worse, broad cooperative connectivity forays with region-wide order-making potential, such as PGII, B3W, or BDN, are currently more aspirational than operational. The current trajectory seems, therefore, to be towards an Indo-Pacific region of incipient connected order*s*, consisting of competing, overlapping, intermeshed connections, and structured around key players, whether the USA, Japan, or China. Other actors can oftentimes, but not always, opt in and out of these orders, an insight that tracks Amitav Acharya’s idea of “multiplexity” [145]. However, such orders are hardly complementary. We can therefore expect a longer period of disarray, where the supply of connectivity does not match the region’s massive demands, and, concomitantly, the prospects for a connected, functioning and sustainable “super-regional” order remain bleak.

## Footnotes

1 Other actors such as Australia and India partially endorsed the FOIP idea, but, as the concept is often seen as a tool to contain China, have not fully embraced it. India, for example, has emphasized the need for inclusivity by coining the term Free, Open and *Inclusive* Indo-Pacific (FOIIP), and maintains the necessary distance and flexibility to tailor its policy choices according to its interlocutor [1:438]. Australia de facto adheres to the FOIP term but does not officially use it [1:424].

2 This fits with the definition provided by the U.S. Indo-Pacific Command, wherein the area “encompasses about half the earth’s surface, stretching from the waters off the west coast of the U.S. to the western border of India, and from Antarctica to the North Pole” [32].

3 The Indo-Pacific is here conceptualized as “a super-region with hard-to-define outer limits and distinct subregions yet an unquestionably Asian core” [48: 473].

4 See: https://www.dfc.gov/who-we-are/overview.

5 Data available at: foreignassistance.gov.
